# Taurine promotes axonal sprouting via Shh-mediated mitochondrial improvement in stroke

**DOI:** 10.1590/acb382323

**Published:** 2023-06-26

**Authors:** Jianwen Jia, Xiaochao Tian, Jinzhao He, Guozhong Ma, Weiliang He

**Affiliations:** 1 Capital Medical University – Beijing Chaoyang Hospital – Department of Neurosurgery – Beijing, China.; 2 Second Hospital of Hebei Medical University – Department of Cardiology – Hebei, China.; 3 Heyuan People’s Hospital – Guangdong Provincial People’s Hospital Heyuan Hospital – Department of Neurology – Guangdong, China.; 4 Heyuan People’s Hospital – Heyuan Key Laboratory of Molecular Diagnosis & Disease Prevention and Treatment – Doctors Station of Guangdong province – Guangdong, China.

**Keywords:** Stroke, Taurine, Mitochondria

## Abstract

**Purpose:**

Motor function is restored by axonal sprouting in ischemic stroke. Mitochondria play a crucial role in axonal sprouting. Taurine (TAU) is known to protect the brain against experimental stroke, but its role in axonal sprouting and the underlying mechanism are unclear.

**Methods:**

We evaluated the motor function of stroke mice using the rotarod test on days 7, 14, and 28. Immunocytochemistry with biotinylated dextran amine was used to detect axonal sprouting. We observed neurite outgrowth and cell apoptosis in cortical neurons under oxygen and glucose deprivation (OGD), respectively. Furthermore, we evaluated the mitochondrial function, adenosine triphosphate (ATP), mitochondrial DNA (mtDNA), peroxisome proliferator-activated receptor gamma coactivator 1-alpha (PCG-1α), transcription factor A of mitochondria (TFAM), protein patched homolog 1 (PTCH1), and cellular myelocytomatosis oncogene (c-Myc).

**Results:**

TAU recovered the motor function and promoted axonal sprouting in ischemic mice. TAU restored the neuritogenesis ability of cortical neurons and reduced OGD-induced cell apoptosis. TAU also reduced reactive oxygen species, stabilized mitochondrial membrane potential, enhanced ATP and mtDNA content, increased the levels of PGC-1α, and TFAM, and restored the impaired levels of PTCH1, and c-Myc. Furthermore, these TAU-related effects could be blocked using an Shh inhibitor (cyclopamine).

**Conclusion:**

Taurine promoted axonal sprouting via Shh-mediated mitochondrial improvement in ischemic stroke.

## Introduction

Stroke is among the primary causes of disability and death worldwide^1^. In the past decade, new research has been focused on brain plasticity including axonal sprouting that is widely recognized in the functional recovery of an injured brain^2^. External interventions have been increasingly reported to accelerate repair processes^3^. Therefore, targeting the promotion of axonal sprouting is a more promising therapeutic strategy for ischemic stroke.

Axonal sprouting requires adenosine triphosphate (ATP) to power fundamental developmental processes^4^. Mitochondria are among the important organelles and produce most of the required ATP^5^. They have been reported to be involved in brain plasticity after brain injury^6^. Several recent studies have established the effects of mitochondria in determining axonal sprouting^7^^-^ 9. Therefore, it may be ideal to identify agents promoting axon regeneration by protecting mitochondria.

Taurine (TAU) is a rich amino acid stored in mammalian brain tissues^10^. It has been considered a target in neurological diseases causing traumatic brain injury, including Alzheimer’s disease, Parkinson’s disease, and ischemic stroke^11^^-^ 15. Studies have indicated that TAU plays a role in neurogenesis^16^^,^ 17. TAU has been reported to protect the brain from experimental stroke by preserving mitochondrial function^18^. Moreover, it exerts potentially protective effects against hepatic encephalopathy, hyperammonemia-induced mitochondrial dysfunction, and energy crisis^19^. However, whether TAU modulates mitochondrial function during axonal sprouting under stroke conditions remains unclear.

Thus, we aimed to assess the role of TAU in axonal sprouting against cerebral ischemic injury, clarify the function of mitochondria in TAU-induced axonal sprouting, and further determine the underlying potential molecular mechanism.

## Methods

This study was supported by grants from the scientific research start-up funds of Heyuan People’s Hospital, the High-level Talent Foundation of Hebei Province in 2021 (No. A202002004), and the National Natural Science Foundation of China (No. 81801312).

The experimental protocols were approved by the Committee of Ethics on Laboratory Animals of Heyuan People’s Hospital and performed according to the recommendations from the Guide for the Care and Use of Laboratory Animals of the National Institute of Health.

### Experimental protocols

**Figure 1:**
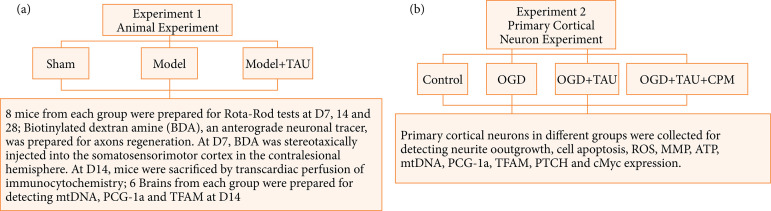
Schematic of experimental design. In **(a)** experiment 1, animals were divided into three groups: sham (0.9% saline), model (0.9% saline), and model + taurine (TAU) (50 mg/kg dissolved in 0.9% saline). In the model + TAU group, TAU (1 mL/kg) was intravenously injected 30 min after the stroke for seven consecutive days. After the stroke, the rotarod test was performed on D7, D14, and D28. On D7 after stroke, biotinylated dextran amine (BDA), an anterograde neuronal tracer, was stereotaxically injected into the somatic sensorimotor cortex in the contralesional hemisphere. The mice were sacrificed by transcardiac perfusion on D14 after TAU treatment. Furthermore, we evaluated mitochondrial function, ATP, mtDNA, the levels of peroxisome proliferator-activated receptor gamma coactivator 1-alpha (PCG-1α), and transcription factor A of mitochondria (TFAM). In **(b)** experiment 2, primary cortical neurons were divided into three groups: control, oxygen and glucose deprivation (OGD), and OGD + TAU groups. We evaluated cell viability and apoptosis, neurite outgrowth, mitochondrial function, ATP, mtDNA, PCG-1α, TFAM, protein patched homolog 1 (PTCH1), and c-Myc levels of different groups. To detect the effect of the Shh pathway, a specific inhibitor of the Shh pathway, cyclopamine (CPM, 2.5 μM in 0.1% dimethyl sulfoxide [DMSO]), was added to the medium 30 min before TAU treatment.

The experiment was randomized into two parts (Fig. 1). Under part 1, we studied the effect of TAU on stroke mice. The mice were randomly divided into three groups:

- Sham: 0.9% saline;
- Model: 0.9% saline;
- Model + TAU: 50 mg/kg dissolved in 0.9% saline.

In the model + TAU group, TAU (1 mL/kg) was intravenously injected 30 min after the stroke for seven consecutive days (D). After the stroke, the rotarod test was performed on D7, D14, and D28. On D7, we injected biotinylated dextran amine (BDA), which was used as an anterograde neuronal tracer, into the contralateral hemispheric somatosensory cortex. On D14, we detected the BDA-labeled axonal density using immunocytochemistry (ICC). The expressions of mitochondrial DNA (mtDNA), peroxisome proliferator-activated receptor gamma coactivator 1-alpha (PCG-1α), and transcription factor A of mitochondria (TFAM) were measured on D14 after stroke.

The role of TAU in primary cortical neurons under oxygen and glucose deprivation (OGD) and the potential underlying mechanism were determined in part 2. The neurons were divided into the following groups:

- Control;
- OGD;
- OGD + TAU.

The levels of neurite outgrowth, cell apoptosis, mitochondrial function, ATP, mtDNA, PCG-1α, TFAM, PTCH1, and c-Myc were detected in different groups. To understand the effect of the Shh pathway, a specific inhibitor of the Shh pathway, cyclopamine (CPM, 2.5 μM in 0.1% dimethyl sulfoxide [DMSO]), was added to the medium 30 min before TAU treatment.

### Focal cerebral cortical ischemia model

According to a previous report, we preceded focal cerebral cortical ischemia in C57BL/6 mice^20^. Briefly, C57BL/6 mice were anesthetized and permanently ligated in the right common carotid artery. Subsequently, the right middle cerebral arteries of the mice were exposed along with the coagulated striatal branch. The abovementioned procedures were performed in all groups. However, coagulation of the striatal branch was excluded in the sham group.

### Rotarod test

The rotarod test was performed on D7, D14, and D28 to assess the motor function. Based on our previous report^3^, we placed the mice on a rotating rotarod cylinder. The speed of the cylinder accelerated from 4 to 40 rpm in less than 300 s. The riding time of the mouse on the cylinder was recorded three times. The mean value was considered for statistical analysis. Each mouse was trained before the tests and rested for 15 min after each test.

### Biotinylated dextran amine injection

After the administration of anesthesia, 10% BDA was injected into the somatosensorimotor cortex of the contralesional hemisphere (two sites/brain, 1 μL/site) of the mice using a stereotactic device with nano injection pump (0.15 μL/min). The coordinates of the two injection sites are as follows:

- 2 mm posterior to the bregma and 1.5 mm lateral to the midline;
- 1.1 mm posterior to the bregma and 1.5 mm lateral to the midline.

Based on a previous study, the needle (1 μL) was kept in situ for 10 min after injection^21^.

### Immunocytochemistry analysis

The brains of mice were blocked with 4% paraformaldehyde. They were then cryoprotected with 30% sucrose and sliced into 20 μm. The three coronal sections, including the somatosensory motor cortex and infarct cavity (+0.74, -0.46, and -1.58 mm from the bregma), were selected for ICC. The sections were fixed with 5% normal goat serum. After adding horseradish peroxidase (1:1,000), the sections were kept overnight at 4 °C. DyLight 633-conjugated goat anti-horseradish peroxidase (1:500, Jackson Immuno Research Labs, United States of America) was added to the cultured sections for 2 h. The nucleus was stained using Hoechst 33342 at a final concentration of 5 μg/mL. The images were obtained using an upright fluorescence microscope (Olympus, Japan).

### The quantification of mtDNA and nuclear DNA

The DNeasy Blood and Tissue kit (Qiagen, Hermantown, MD, United States of America) was used to extract total DNA. Real-time polymerase chain reaction (RT-PCR) (Applied Biosystems, United States of America) was performed in the presence of SYBR Green I (CWBIO, China) to detect the copy number of the mtDNA. Compared with nuclear DNA (rRNA 18S), the relative copy number of mtDNA was detected. The primers used for mtDNA and rRNA 18S quantification are as follows^22^:

mtDNA: sense, 5’-GCCCCAGATATAGCATTCCC-3’;

anti-sense, 5’-GTTCATCCTGTTCCTGCTCC-3’;

rRNA 18S: sense, 5’-TAGAGGGACAAGTGGCGTTC-3’;

anti-sense, 5’-CGCTGAGCCAGTCAGTGT-3’.

### Quantitative real-time polymerase chain reaction

Trizol reagent (Invitrogen, United States of America) was used to extract total RNA, and the first-strand cDNA synthesis kit (Fermentas International Inc, Canada) was used to reverse-transcribe the RNA into cDNA. In the presence of SYBR Green I (CWBIO, China), the cDNA was amplified using the RT-PCR system (Applied Biosystems company, USA). The 2^–∆∆CT^ method was performed to calculate relative PCR products with the mouse glyceraldehyde 3-phosphate dehydrogenase (GAPDH) gene as the control. The related gene sequences for quantitative RT-PCR (RT-qPCR) are as follows^23^^,^ 24:

PCG-1α: Forward, 5’-CACCAA ACCCACAGAAAACAG-3’;

Reverse, 5’-GGGTCAGAGGAAGAGATAAAGTTG-3’;

TFAM: Forward, 5’-CACCCAGATGCAAAACTTTCAG-3’;

Reverse, 5’-CTGCTCTTTATACTTGCTCACAG-3’;

PTCH1: Forward, 5’-ACCCGCCAGAAGATAGGAGA-3’;

Reverse: 5’-GGAGTGCTGAGTCCAGGTGT-3’;

c-Myc: Forward, 5’-ATCAAGAGGCCACAGCAAAC-3’;

Reverse, 5’-TTGGCAGCTGGATAGTCCTT-3’;

GAPDH: Forward, 5’-GACATCATACTTGGCAGG-3’;

Reverse, 5’-CTCGTGGAGTCTACTGGT-3’.

### Primary cortical neuron culture and oxygen and glucose deprivation

The cerebral cortex tissues from the embryonic brains (D18) of C57BL/6 were removed. The dispersed tissues were dissociated into a single-cell mixture using Hibernate-E (Sigma, United States of America). Cells were plated onto culture dishes (BD Biosciences, United States of America) coated with poly-L-lysine and grown in neurobasal medium with 2% B-27 (Invitrogen, United States of America) and 0.5 mM glutamine (Life Technologies, United States of America) at 37 °C in a 5% CO_2_ incubator. OGD was used to initiate ischemia. We used the same incubator in combination with a hypoxic workstation containing a gas mixture of 0.1% O_2_, 94.9% N_2_, and 5% CO_2_. To detect the effect of the Shh pathway, a specific inhibitor of the Shh pathway, CPM (2.5 μM in 0.1% DMSO), was added to the medium 30 min before TAU treatment.

### Neurite outgrowth and apoptosis

To measure the length of neurite outgrowth of the cortical neurons, cells were stained with mouse monoclonal anti-β III tubulin antibody (Tuj-1, 1:500, Sigma, United States of America) using the immunofluorescence method. Tuj1-positive cells were captured at ×40 objective with an upright fluorescence microscope (Olympus, Tokyo, Japan). The longest neurite length of Tuj-1-positive cell (60/group) was measured using the Image-J analysis system.

The apoptosis of neurons was detected by staining the cells with Hoechst 33342 at the final concentration of 5 μg/mL. Cells were observed immediately using a fluorescence microscope. Additionally, apoptotic cells were also detected using an Annexin V-FITC/PI apoptosis detection kit according to the manufacturer’s protocols. Apoptotic and necrotic cells were quantified using a FACSCalibur cytometer (Becton Dickinson, Franklin Lakes, New Jersey, United States of America). The rate of apoptosis was determined using the Cell Quest software.

### Determination of reactive oxygen species

The intracellular *reactive oxygen species (*ROS) level was detected using 2, 7-Dichlorofluorescin diacetate (DCFH-DA, Beyotime, China), as previously described^3^. Briefly, after the treatment, cells were incubated with DCFH-DA at 37 °C for 30 min and then washed with phosphate buffered saline. The fluorescence intensity was then immediately detected using a luminescence spectrometer (excitation: 488 nm and emission: 525 nm, LS50B, PerkinElmer, United States of America). Data are presented as percent of control.

### Determination of mitochondrial membrane potential

The JC-1 assay kit (Beyotime, China) was used to detect the changes in mitochondrial membrane potential (MMP). In brief, cells were collected and stained with JC-1 staining solution (5 μg/mL) for 20 min at 37 °C and rinsed with JC-1 staining buffer twice. The fluorescence intensity of JC-1 was detected using a multimode reader (Tecan, Switzerland). Data were presented as a percentage of control. In addition, fluorescence images of MMP were obtained using a fluorescence microscope.

### Adenosine triphosphate detection

The ATP levels were detected using an ATP assay kit (Beyotime, China) according to the manufacturer’s protocols. Luminance intensity was measured using a monochromatic microplate reader (Tecan, Switzerland). Data were presented as percent of control. The ATP content was determined as a percentage of untreated cells (control).

### Statistical analysis

Quantitative data were presented as mean ± standard error of measurement (SEM). Statistical analysis was performed using the Statistical Package for the Social Sciences (SPSS) 17.0 software. One-way analysis of variance (ANOVA) was performed for statistical analysis, and then Turkey’s post hoc test or Student t-test was used. A *P* < 0.05 was considered statistically significant.

## Results

### Taurine improves recovery of motor function in stroke

**Figure 2:**
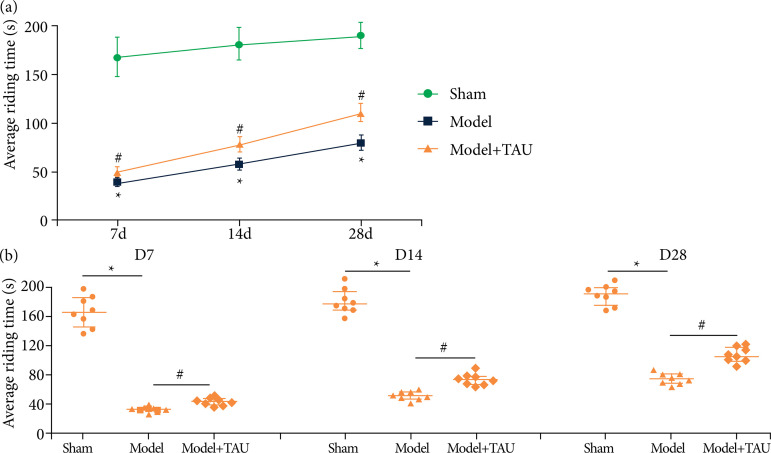
Motor recovery in the different groups in stroke mice. Data revealed that DL-3-n-butylphthalide (NBP) significantly improved motor recovery. **(a** and **b)**. Rotarod test on D7, D14, and D28 after stroke. The results are shown as the mean ± standard error of the mean (n = 8).

To observe the effect of TAU on the motor function of stroke, the rotarod test was performed. Compared with the sham group, the average riding time of the model group on D7, D14, and D28 was shorter (*P* < 0.05). However, mice were alive for more time in the TAU-treated groups compared with those in the model groups (*P* < 0.05), indicating that TAU could improve the impairments in the motor function induced by ischemic stroke (Fig. 2).

TAU: taurine; *P < 0.05 vs. the sham group; ^#^ *P* < 0.05 vs. the model group.

### Taurine promotes axonal sprouting in ischemic stroke

**Figure 3:**
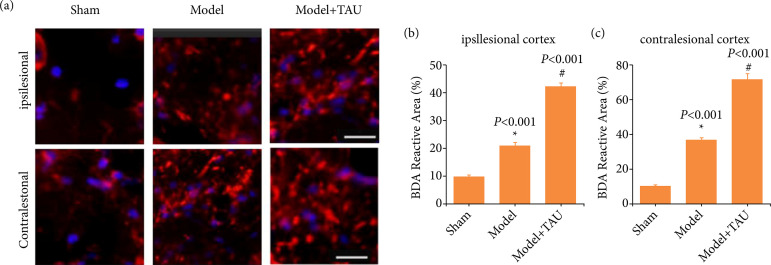
Effect of taurine (TAU) on axonal sprouting in ischemic stroke. **(a)** Representative images of the biotinylated dextran amine (BDA)-labeled axons in the contralesional cortex and the ipsilesional cortex; scale = 50 μm. **(b)** The density of BDA-labeled axons in the ipsilesional cortex. **(c)** The density of BDA-labeled axons in the contralesional cortex. The results are shown as the mean ± standard error of the mean (n = 6).

We further explored the potential mechanism of TAU on axonal sprouting in stroke. As shown in Fig. 3, a few BDA-labeled axons could be observed in the sham group of the ipsilesional cortex. The axonal density of BDA-labeled axons was greater in the model group than in the sham group. However, the highest density was observed in the model + TAU group. Interestingly, the same trend was observed in the contralesional cortex. These results suggested that TAU could promote the growth of axonal branches in the ipsilateral cortex, as well as the contralateral cortex.

*P < 0.05 vs. the sham group; ^#^ *P <* 0.05 vs. the model group.

### Taurine restores mitochondrial-related factors of the brain after an ischemic stroke

**Figure 4:**
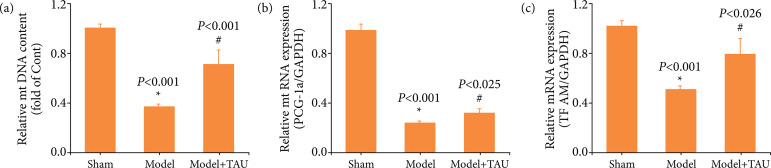
Effect of taurine (TAU) on mitochondria-related factors after an ischemic stroke. **(a)** The mitochondrial DNA (mtDNA) content was measured in different groups. **(b)** The mRNA level of peroxisome proliferator-activated receptor gamma coactivator 1-alpha (PCG-1α) was measured by quantitative real-time polymerase chain reaction (RT-qPCR). **(c)** The mRNA level of transcription factor A of TFAM was measured by RT-qPCR. Results are expressed as the mean ± standard error of the mean (n = 6).

To explore the underlying mechanisms, we focused on the mitochondria, and the expression of mitochondrial biogenesis markers was estimated. The mtDNA content significantly decreased in the model group, but increased after TAU treatment. Additionally, TAU significantly increased the expression of PGC-1α, and TFAM after stroke (Fig. 4). The data showed that TAU could preserve the mitochondria-related factors after an ischemic stroke.

*P < 0.05 vs. sham group; ^#^ *P* < 0.05 vs. model group.

### Taurine restores the neuritogenesis ability of cortical neurons induced by oxygen and glucose deprivation

**Figure 5:**
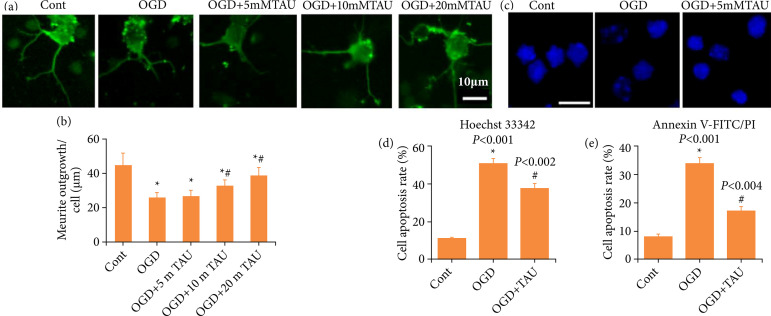
Effect of taurine (TAU) on neurite outgrowth and apoptosis in primary cortical neurons under oxygen and glucose deprivation (OGD). **(a)** Neurons were stained with the anti-β-III-tubulin (Tuj-1) antibody. Representative images of Tuj-1-positive neurons in different conditions. Scale = 10 μm. **(b)** Quantitative analysis of the length of the longest neurite. Values are expressed as the mean ± standard error of the mean (SEM) (n = 60); *P < 0.05 vs. control group; ^#^ *P* < 0.05 vs. OGD group. **(c and d)** The apoptotic rate was determined by Hoechst 33342 staining. **(e)** The apoptotic rate was determined by using the Annexin V-FITC/PI apoptosis detection kit. Values are expressed as the mean ± SEM (n = 6); *P < 0.05 vs. control group; ^#^ *P* < 0.05 vs. OGD group.

We further explored the effect of TAU on the neurite outgrowth of cortical neurons under OGD. As shown in Figs. 5a and 5b, compared with the control group, the length of neurite outgrowth in the OGD group showed remarkable shortening. However, in the presence of different concentrations of TAU (10/20 mM), the length of neurite outgrowth significantly recovered. These results indicated that 20 mM TAU could significantly recover neuronal neuritogenesis ability after an ischemic injury, and this concentration was used for subsequent experiments. Additionally, the apoptotic rate was determined. As shown in Figs. 5c and 5d, the apoptotic rates in the control, OGD, and OGD + TAU groups were 11.39 ± 1.41%, 51.08 ± 5.80%, and 38.27 ± 5.21%, respectively. The Annexin V-FITC/PI apoptosis detection results also indicated that TAU reduced apoptosis induced by OGD (Fig. 5e). All these results suggested that TAU could protect cortical neurons against ischemic injury.

### Taurine preserves mitochondria-related factors in cortical neurons under oxygen and glucose deprivation

**Figure 6:**
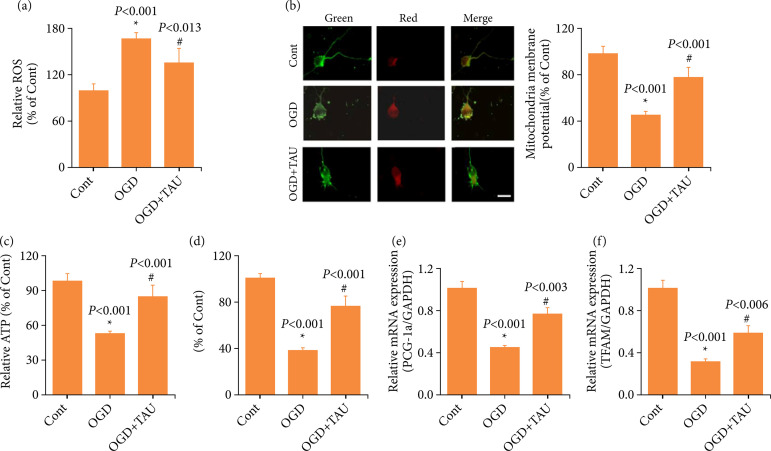
Effect of taurine (TAU) on mitochondria-related factors in primary cortical neurons under oxygen and glucose deprivation (OGD). **(a)** Generation of reactive oxygen species (ROS) in different groups. **(b)** Mitochondrial membrane potential (MMP) was determined by JC-1 staining under an upright fluorescence microscope. **(c)** Cellular adenosine triphosphate (ATP) concentrations were assessed in different groups. **(d)** The mtDNA content was measured in different groups. **(e and f)** The mRNA levels of PCG-1α and transcription factor A of TFAM were measured by quantitative real-time polymerase chain reaction (RT-qPCR). Results are expressed as the mean ± standard error of the mean (n = 6).

We investigated the potential mechanism of TAU-induced protection. Firstly, mitochondrial function was examined. Neurons under OGD exhibited ROS accumulation and dissipated MMP. In neurons incubated with TAU, ROS accumulation was reduced (Fig. 6a), and MMP was significantly restored (Fig. 6b). Secondly, ATP levels were assessed. TAU significantly increased the ATP levels, which were reduced compared to those in the OGD group (Fig. 6c). Lastly, under OGD, the mtDNA content significantly decreased, but increased after TAU treatment (Fig. 6d). Additionally, TAU significantly upregulated the expression of PGC-1α and TFAM after neurons were under OGD (Figs. 6e and 6f). The above data indicated that TAU preserved the mitochondria-related factors in cortical neurons under OGD.

*P < 0.05 vs. control group; ^#^ *P* < 0.05 vs. OGD group.

### The Shh pathway is involved in the protective effect of taurine on the mitochondria in cortical neurons under oxygen and glucose deprivation

**Figure 7:**
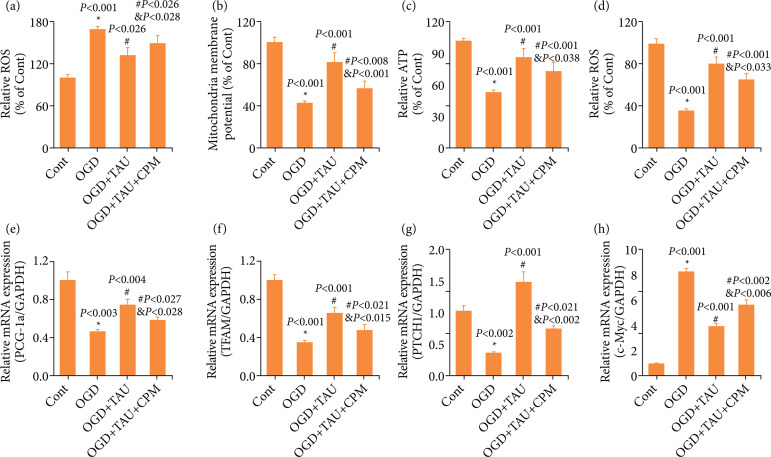
The Shh pathway involving in the neuroprotective effect of taurine (TAU) on mitochondria-related factors in cortical neurons under oxygen and glucose deprivation (OGD). Effect of cyclopamine (CPM), a specific inhibitor of the Shh pathway, on the TAU-induced improvement of mitochondria were assessed by measuring **(a)** reactive oxygen species (ROS) levels, **(b)** mitochondrial membrane potential (MMP), **(c)** adenosine triphosphate (ATP) levels, **(d)** mitochondrial DNA (mtDNA) content, **(e)** the mRNA levels of proliferator-activated receptor gamma coactivator 1-alpha (PCG-1α), **(f)** transcription factor A of mitochondria (TFAM), **(g)** the mRNA levels of protein patched homolog 1 (PTCH1), and **(h)** cellular myelocytomatosis oncogene (c-Myc). Data are presented as mean ± standard error of the mean (n = 6).

In our previous study, we found that Shh exerted a neuroprotective effect and caused neurite outgrowth of cortical neurons by preventing mitochondrial dysfunction^25^. To further study the underlying mechanism, we explored whether the Shh pathway was involved in this process. Firstly, we administrated CPM to observe the mitochondrial functions. Compared with the TAU group, CPM significantly boosted ROS (Fig. 7a), reduced MMP (Fig. 7b), and decreased ATP levels (Fig. 7c). Secondly, we determined the effect of CPM on mitochondrial biogenesis in cortical neurons under OGD. Compared with the TAU group, CPM partly decreased the mtDNA content (Fig. 7d) and reduced the levels of PGC-1α and TFAM (Figs. 7e and 7f). Lastly, we tested the effect of TAU on the Shh pathway and CPM treatment by analyzing the expression of the Shh pathway genes such as PTCH1 and c-Myc. CPM partly reduced the levels of PTCH1 and c-Myc that TAU restored in neurons injured by OGD (Fig. 7 g-h). Collectively, these results indicated that TAU improved the mitochondrial function in cortical neurons under OGD possibly through the Shh pathway.

*P < 0.05 vs. control group; ^#^ *P* < 0.05 vs. OGD group; ^&^ *P* < 0.05 vs. OGD + TAU group.

## Discussion

In the present study, we determined that TAU improved motor function recovery and restored neurogenesis in ischemic stroke. This possibly occurred via improvements in mitochondrial function. Furthermore, we investigated that the Shh pathway exerted an important role in these effects. Our study findings highlighted the novel viewpoint that TAU promoted axonal sprouting by improving Shh-mediated mitochondrial function in cerebral ischemic stroke.

It is important to recover motor function among disabled patients with stroke^21^. A study reported that brain plasticity, including axonal spouting, is positively associated with motor function recovery^26^. Therefore, it is imperative to identify a novel pharmaceutical for promoting axonal sprouting in stroke. TAU is the main intracellular free-amino acid present in most animal tissues (including the brain). It can pass through the blood–brain barrier and exerts a protective role after brain ischemia^18^.

Indeed, several studies have reported that TAU plays a protective role in stroke^27^^-^ 30. A study reported that TAU can be used to experimentally treat neuronal damage^31^. Schurr et al.^32^ suggested that TAU pretreatment can restore synaptic function in rat hippocampal slices. Furthermore, TAU increased the survival rate of newborn neurons and improved neurogenesis in adults^16^. However, whether TAU affects axonal sprouting in ischemic stroke remains unclear. Our results suggested that TAU revived motor function and promoted axonal sprouting after stroke.

The mechanism by which TAU improves axon sprouting has been further studied. The mitochondria, including axon remodeling, may exert a crucial effect on controlling neuroplasticity. Impaired mitochondria may impair neural plasticity after stroke^6^. Recently, a study reported that neurological diseases might be related to mitochondrial biogenesis^33^. Another study reported that, after cerebral ischemia, biological damage might adversely affect mitochondrial function in vitro^34^. Preservation of mtDNA copy number may alleviate damage to mitochondrial biogenesis^35^. We observed that loss of mtDNA induced by insult injury was restored after TAU treatment. In addition, PGC-1α could upregulate the expression of genes related to mitochondrial biology or increase their transcription activity^36^. TFAM regulated the copy number of mtDNA^37^. We measured the levels of TFAM and PGC-1α and observed that TAU could restore ischemic injury-induced increases in their levels. Taken together, the data suggested that TAU-induced axonal spouting after stroke was partially mediated by enhanced mitochondrial function.

We also investigated the potential molecular mechanisms underlying mitochondrial function in TAU-stimulated axonal spouting. A study reported that the Shh pathway is involved in axonal spouting^38^. Shh overexpression increased neurogenesis in the dentate gyrus of nonischemic rats, but its blockade using CPM abolished cerebrolysin-induced neurogenesis in adult rats^39^^,^ 40. In cortical neurons, Shh may have improved neurite outgrowth by preventing mitochondrial dysfunction^25^^,^ 41. Furthermore, in mice, TAU promoted the proliferation and differentiation of cochlear neural stem cells via the Shh pathway^42^. However, whether Shh mediates the effect of TAU on the mitochondria in axonal remodeling remains unknown. Therefore, we hypothesized that TAU improves mitochondrial function in the axon via the Shh pathway. Interestingly, inhibition of the Shh pathway with CPM exacerbated the levels of ROS, MMP, ATP, mtDNA, PCG-1α, TFAM, PTCH1, and c-Myc, which were improved by TAU in neurons under OGD. Taken together, these data suggested that TAU-induced mitochondrial improvements in neurons partially occurred via Shh pathway.

Our study has some limitations. First, additional studies are warranted to elucidate the detailed mechanisms by which TAU in axonal spouting regulates the mitochondria to prevent focal cerebral ischemia; this is beneficial in understanding TAU modulation. Furthermore, the additional mechanisms underlying the role of TAU in axonal spouting after ischemia stroke, such as the PI3K/Akt, MAPK/Erk, and other pathways, need to be explored further.

## Conclusions

Our study results suggested that TAU promoted axonal spouting after stroke via mitochondrial regulation. This protective effect was partially mediated by the Shh pathway. Our findings highlighted that TAU might contribute to the therapeutic intervention of axonal remodeling after ischemic stroke-targeted mitochondrial and Shh pathways.

## Data Availability

All generated data were presented in this study.
